# Patient Perceptions of Surgeon Reimbursement in Total Ankle Arthroplasty

**DOI:** 10.1177/19386400231183602

**Published:** 2023-07-14

**Authors:** Austin B. Mike-Mayer, Vishaal Sakthivelnathan, Akhil Sharma, Vinod K. Panchbhavi, Jie Chen

**Affiliations:** Department of Orthopedic Surgery and Rehabilitation, The University of Texas Medical Branch, Galveston, Texas; John Sealy School of Medicine, The University of Texas Medical Branch, Galveston, Texas; St. Luke’s University Hospital, Bethlehem, Pennsylvania; Department of Orthopedic Surgery and Rehabilitation, The University of Texas Medical Branch, Galveston, Texas; Department of Orthopedic Surgery and Rehabilitation, The University of Texas Medical Branch, Galveston, Texas

**Keywords:** affordable care act, ankle replacement arthroplasty, health care costs, Medicare, orthopedic surgeons, reimbursement, total ankle replacement

## Abstract

Introduction. The incidence of total ankle arthroplasty (TAA) for ankle osteoarthritis has increased in the Medicare population by approximately 16.37% each year. This study examines the patient perception of orthopedic surgeon reimbursement for TAA by Medicare. Methods. A total of 78 patients were surveyed anonymously at 2 foot and ankle clinics within an academic health care setting. The surveys were given anonymously before the patients were seen by an orthopedic surgeon. Surveys were returned to office staff who placed them in a collection box to ensure confidentiality. Results. The average estimate of how much orthopedic surgeons should be reimbursed for TAA was $19 506 and the average estimate of how much orthopedic surgeons were actually reimbursed was $20 772. Fifty patients believed that orthopedic surgeons were under reimbursed, 9 believed that they were reimbursed appropriately, and 19 were unsure. Demographic variables such as age, sex, education level, income, and insurance status had no significant effect on the results. Conclusions. Most patients believed orthopedic surgeons are under reimbursed for TAA and that there is a lack of health care transparency regarding orthopedic reimbursement for TAA by Medicare.

**Levels of Evidence:** Level V: Expert opinion

“Total ankle arthroplasty (TAA) for ankle osteoarthritis is a procedure in which the ankle joint is removed and replaced with an implant.”

## Introduction

Total ankle arthroplasty (TAA) for ankle osteoarthritis is a procedure in which the ankle joint is removed and replaced with an implant. The utilization of TAA is increasingly popular among surgeons; from 2005 to 2012, the reported incidence of TAA within the Medicare population was 7181, with a compound annual growth rate of 16.37%.^1^

Medical economics and health care transparency have become more important over the past decade. With increasing health care costs in the United States, changes implemented after the Affordable Care Act was passed have resulted in decreased reimbursements for orthopedic surgeons,^2^ which has, in turn, led to physicians re-evaluating the cost-effectiveness of accepting Medicare patients.

Studies regarding patient perception of total knee, hip, and shoulder arthroplasty reimbursements revealed that patients overestimated Medicare reimbursement and believed that orthopedic surgeons are under reimbursed.^3-5^ However, there are no similar studies regarding TAA. This study surveyed patients at 2 foot and ankle clinics to determine patient understanding of TAA Medicare reimbursement and assess whether they believe orthopedic surgeons performing TAA are compensated appropriately. We hypothesize that patients overestimate the amount that orthopedic surgeons are reimbursed for TAA.

## Materials and Methods

A survey was distributed to 100 patients who came into 2 foot and ankle clinics. The surveys were given anonymously and were returned to the office staff before the patients were seen by an orthopedic surgeon. Surveys were then placed in a collection box to ensure confidentiality.

The survey consisted of an introduction and 3 parts (see Appendix). The introduction provides information on TAA indications and benefits and included the purpose of this study. Part 1 included demographic questions about age, sex, history of TAA, education level, annual household income, and medical insurance status. Part 2 asked questions regarding the patient’s opinion of TAA reimbursement before they were told the average reimbursement rate. Questions in part 2 included the following:

(1) How much do you believe an orthopedic surgeon should be reimbursed for a TAA?
(2) How much do you believe Medicare reimburses orthopedic surgeons for a TAA and care 90 days after the surgery?

Part 3 revealed the average reimbursement for TAA ($1018) and asked the following follow-up question:

(1) Do you believe that orthopedic surgeons are _______ for TAA and postop care 90 days after the surgery?
  (a) Under reimbursed
  (b) Over reimbursed
  (c) Reimbursed appropriately

The data were consolidated into an Excel sheet. Chi-squared tests for significance were used to determine whether any of the demographic variables had a significant effect on our primary outcome (patient perception of orthopedic surgeon reimbursement for TAA).

## Results

**Figure 1.**
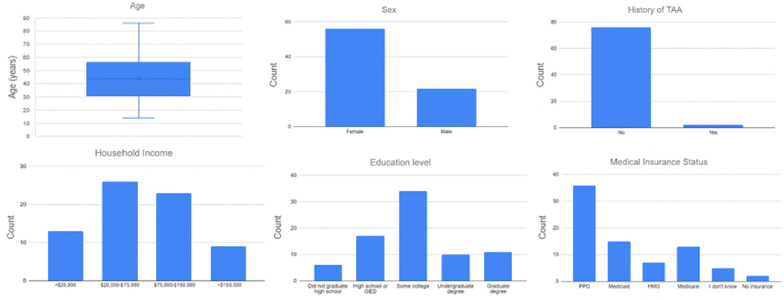
Demographic variable distributions.

A total of 78 surveys were completed. Table 1 and Figure 1 report the distribution of the demographic variables and their significance in relation to our primary outcome. Respondent ages ranged from 18 to 86 years old, with an average age of 42 and a standard deviation of 17 years. Table 2 reports the distribution of answers patients gave as to whether they thought orthopedic surgeons were under reimbursed, reimbursed appropriately, over reimbursed, or still unsure regarding Medicare reimbursement for TAA.

**Table 1.** Distribution of Demographic Variables and Their Significance (*P*) in Relation to Primary Outcome.

Demographic variable	Number of responses	Significance (*P*)
Sex		0.93
Female	56	
Male	22	
History of TAA		0.56
Yes	2	
No	78	
Education level		0.31
Did not graduate high school	6	
High school or GED	17	
Some college	34	
Undergraduate degree	10	
Graduate degree	11	
Household income		0.76
<$20 000/year	13	
$20 000-$75 000/year	26	
$75 000-$150 000/year	23	
>$150 000/year	9	
Medical insurance		0.34
Medicare	13	
Medicaid	15	
HMO	7	
PPO	36	
Uninsured	2	
Not sure	5	

Abbreviations: GED, graduate equivalency degree; HMO, health maintenance organization; PPO, preferred provider organization; TAA, total ankle arthroplasty.

**Table 2.** Perception of Orthopedic Surgeon Reimbursement for Total Ankle Arthroplasty by Medicare.

Patient perception	Number of responses
Under reimbursed	50
Reimbursed appropriately	9
Over reimbursed	0
Unsure	19

Overall, 64% of patients believed that orthopedic surgeons were under reimbursed, 12% believed that the reimbursement amount was appropriate, 0% believed that orthopedic surgeons were over reimbursed, and 24% were still undecided.

We also asked how much patients thought orthopedic surgeons should be reimbursed before they were made aware of the actual amount. Nine patients responded to this question. The responses ranged from $4000 to $100 000, with a mean of $19 506 and a standard deviation of $31 537. In addition, we asked how much patients thought orthopedic surgeons were reimbursed by Medicare before they were made aware of the actual amount. The same 9 patients responded to this question, and the responses ranged from $500 to $120 000, with a mean of $20 772 and a standard deviation of $38 535.

## Discussion

The Affordable Care Act passed in 2010 sought to increase access to care while supporting innovation that would lead to decreased health care costs. Nevertheless, health care costs have risen from $2.6 trillion in 2010 to $4.2 trillion in 2020 while reimbursements for orthopedic surgeons have been declining.^6^ This has resulted in providers re-evaluating whether they will accept Medicare or Medicaid as a form of payment. A 2021 survey of orthopedic surgeons revealed that 22% of respondents were either not sure or would not accept Medicare or Medicaid as a form of payment.^7^ Patients’ awareness of orthopedic surgeon reimbursement for TAA has not been researched. We found that a majority of patients believed orthopedic surgeons were under reimbursed for TAA, and that patient perception of reimbursement was not significantly affected by age, sex, education level, income, or insurance status.

Patient awareness of Medicare reimbursement has been examined in other orthopedic procedures, such as total knee arthroplasty, total hip arthroplasty, rotator cuff repairs, and total shoulder arthroplasty. Foran et al^3^ reported that, on average, patients believed that orthopedic surgeons should be reimbursed $14 358 for total hip arthroplasty, and 68.5% of patients believed that the actual reimbursement of $1375 was low. When asked about total knee arthroplasty, patients thought that reimbursement for the procedure was $13 332, and 67.2% of patients believed that the actual reimbursement of $1450 was low. Similar to this study, they found that education level, household income, and insurance status had no significant effect on patients’ perceived reimbursement rates for orthopedic surgeons performing total hip or total knee arthroplasty. A study by Nagda et al^5^ found that patients believed orthopedic surgeons should be reimbursed $8459 for rotator cuff repair and that 75% of patients believed that the actual reimbursement of $1172 was low. Patients also thought that orthopedic surgeons should be reimbursed $13 178 for total shoulder arthroplasty, and 87% of patients believed that surgeons with advanced shoulder training should be reimbursed more. These results are similar to those from this study.

Another important finding from this study was that a majority of patients felt they did not know enough about the health care system to make an educated guess on orthopedic surgeon reimbursement for TAA. A total of 69 out of the 78 patients surveyed left the questions regarding their initial estimate of orthopedic surgeon reimbursement blank or wrote comments such as “I have no idea about how much this procedure costs” or “I do not understand how health care costs are billed.” Only 9 out of the 78 patients surveyed provided an estimate of how much they believe orthopedic surgeons should be reimbursed and how much orthopedic surgeons were actually reimbursed. Both the wide range of estimated reimbursements and the majority of responses with no estimates suggest that there is a lack of transparency regarding TAA reimbursements and health care costs in general.

There are limitations to this study. First, our survey was limited to 2 clinics that were in a close geographical range. Thus, the ability to generalize the results to the entire US population may not be strong. In addition, the format and distribution of the survey could have affected the results. First, the questions are meant to be answered in a certain order. The patients are only meant to be made aware of actual reimbursement after they give their estimates. We made the final question a separate page at the end of the survey so that patients would make their estimates of TAA reimbursement before knowing the actual amount. However, it is possible that patients saw the final page as they were flipping through the survey before they provided their own estimates. In addition, the surveys were given while patients were waiting to see their providers. Thus, if they had a favorable view of their provider, they may have responded with a higher reimbursement estimate. Along the same lines, if they had an unfavorable view of their provider, they may have responded with a lower estimate. Finally, TAA is not as commonly performed as total hip arthroplasty, total knee arthroplasty, rotator cuff repair, or total shoulder arthroplasty, as examined by the Foran et al^3^ and Nagda et al^5^ studies. Only 2 out of the 78 patients surveyed had a TAA before. Therefore, it is possible that the high frequency of “I do not know” responses could be because patients were not aware of the procedure. In order to mitigate this, we included a section on the first page that explained the indications and outcomes of TAA.

## Conclusions

Overall, we found that most patients either believe that orthopedic surgeons are under reimbursed for TAA by Medicare or do not know enough background of the Medicare reimbursement model to make an educated decision. The lack of transparency is also an explanation of the disconnect patients perceive between rising health care costs and the prevalent assumption that the rise is mainly attributed to physician reimbursement rate increases. This study reinforces the necessity of increased health care transparency for better patient awareness and decision-making. Our data can also aid legislation regarding health care policy so that orthopedic surgeons can provide better care for patients needing a TAA.

## Supplemental Material

sj-docx-1-fas-10.1177_19386400231183602 – Supplemental material for Patient Perceptions of Surgeon Reimbursement in Total Ankle Arthroplasty Supplemental material, sj-docx-1-fas-10.1177_19386400231183602 for Patient Perceptions of Surgeon Reimbursement in Total Ankle Arthroplasty by Austin B. Mike-Mayer, Vishaal Sakthivelnathan, Akhil Sharma, Vinod K. Panchbhavi and Jie Chen in Foot & Ankle Specialist
