# Supplementary material for: Patient Perceptions of Surgeon Reimbursement in Total Ankle Arthroplasty
Source: Foot Ankle Spec. 2023 Jul 14;18(3):258–62. doi: 10.1177/19386400231183602 (PMC12064855; doi:10.1177/19386400231183602)
Supplement: sj-docx-1-fas-10.1177_19386400231183602 – Supplemental material for Patient Perceptions of Surgeon Reimbursement in Total Ankle Arthroplasty [file sj-docx-1-fas-10.1177_19386400231183602.docx]

Total Ankle Replacement Survey

Ankle arthritis is a condition in which inflammation of the ankle joint causes pain, stiffness, and difficulty walking or putting weight on the ankle. Total Ankle Replacement (TAR) is a surgical technique used as an option to treat ankle arthritis. TAR replaces the damaged ankle joint with an implant. Most patients report that they noticed significant improvements in pain and ability to walk. At around 4 months after the surgery, patients usually say they feel better than before surgery and feel the most benefits at around 1 year after the surgery. Data from a recent study shows that almost 90% of implants last at least 10 years if not longer.

The purpose of this survey is to understand the perception of Medicare reimbursement for the TAR procedure. Survey results are confidential. The provider will be unaware of completion of the survey.

1. Age:
2. Sex:
3. Have you ever had a TAR before?
4. Education level (select one)
   1. Did not graduate high school
   2. High school or GED
   3. Some college
   4. Undergraduate degree
   5. Graduate degree
5. Annual household income (select one) a. <$20,000

b. $20,000-$75,000

c. $75,000-$150,000

d. >$150,000

1. Medical insurance status (select one)
   1. I don’t know
   2. Medicare
   3. Medicaid
   4. HMO
   5. PPO
2. How much do you believe an orthopedic surgeon should be reimbursed for a TAR:
3. How much do you believe Medicare reimburses orthopedic surgeons for a TAR and care 90 days after the surgery:

The average reimbursement by Medicare for a TAR is $1018

1. Do you believe that orthopedic surgeons are for TAR and postop care 90 days after surgery
   1. Under reimbursed
   2. Over reimbursed
   3. Reimbursed appropriately
2. Please add any additional comments here
